# Supplementary material for: Multiomic analysis of the Arabian camel (Camelus dromedarius) kidney reveals a role for cholesterol in water conservation
Source: Commun Biol. 2021 Jun 23;4:779. doi: 10.1038/s42003-021-02327-3 (PMC8222267; doi:10.1038/s42003-021-02327-3)
Supplement: Supplementary file 2 — Supplementary Information [file 42003_2021_2327_MOESM2_ESM.pdf]

Multiomic analysis of the Arabian camel (*Camelus dromedarius*) kidney reveals a role for cholesterol in water conservation

Fernando Alvira-Iraizoz[1], Benjamin T. Gillard[1*], Panjiao Lin[1], Alex Paterson[1], Audrys G. Pauža[1], Mahmoud A. Ali[2], Ammar H. Alabsi[3], Pamela A. Burger[4], Naserddine Hamadi[5], Abdu Adem[2,6†], David Murphy[1†], Michael P. Greenwood[1†]*

[1]Molecular Neuroendocrinology Research Group, Bristol Medical School: Translational Health Sciences, University of Bristol, Dorothy Hodgkin Building, Bristol, United Kingdom.

[2]Department of Pharmacology and Therapeutics, College of Medicine & Health Sciences, United Arab Emirates University, AL Ain, United Arab Emirates.

[3]College of Medicine, Alfaisal University, Riyadh, Saudi Arabia.

[4]Department of Interdisciplinary Life Sciences, Research Institute of Wildlife Ecology, Vetmeduni Vienna, Vienna, Austria.

[5]Department of Life and Environmental Sciences, College of Natural and Health Sciences, Zayed University, Abu Dhabi, United Arab Emirates.

[6]Department of Pharmacology and Therapeutics, College of Medicine & Health Sciences, Khalifa University, Abu Dhabi, United Arab Emirates.

[*]These authors contributed equally

[†]These authors jointly supervised this work

Corresponding authors:

Fernando Alvira-Iraizoz (f.alvirairaizoz@gmail.com)

Abdu Adem (abdu.adem@ku.ac.ae)

Supplementary material

Supplementary Table ST1. RNAseq baseMean comparison. baseMean comparison (median, mean, maximum and minimum) between all transcripts detected in the medulla and the cortex. These parameters are also shown for transcripts that were detected in both transcriptomes and proteomes and for those only detected in the transcriptomes. baseMean Median and mean were higher in the medulla than in the cortex. It is worth noting that only the more expressed transcripts were detectable at both RNA and protein level as depicted by the lower median and mean for transcripts detected in both transcriptomes and proteomes.

	Median	Mean	Max	Min
Medulla (All)	155.0786	886.8889	358096.8	0.0544388
Medulla (PROT/RNA detectable overlap)	1015.4245	2310.2026	358096.8	0.0544388
Medulla (RNA detectable only)	47.42056	337.8154	49501.34	0.0544388
Cortex (All)	76.18482	770.068	488256.1	0.01360063
Cortex (PROT/RNA detectable overlap)	666.25812	2016.088	488256.1	0.01360063
Cortex (RNA detectable only)	19.20731	232.4503	44562.56	0.01360063

Supplementary Table ST2. RNAseq and proteomic data. Differential gene and protein expression of enzymes involved in the cholesterol biosynthesis pathway, ion transporters and AQP2 in the one-humped Arabian camel kidney. Pairwise comparisons between control, dehydration and dehydration are shown for both cortex and medulla. (UP-REG* - significantly upregulated, DOWN-REG* - significantly downregulated, Non-Sig – non-significantly changed, NA – not detected by mass spectrometry; Benjamini-Hochberg $p_{adj} < 0.05$ was considered significant).

	Arabian Camel											
	RNA						PROT					
	Dehydrated vs Control		Rehydrated vs Dehydrated		Rehydrated vs Control		Dehydrated vs Control		Rehydrated vs Dehydrated		Rehydrated vs Control	
	CORTEX	MEDULLA	CORTEX	MEDULLA	CORTEX	MEDULLA	CORTEX	MEDULLA	CORTEX	MEDULLA	CORTEX	MEDULLA
**ACAT2**	Non-Sig	DOWN-REG*	Non-Sig	UP-REG*	Non-Sig	DOWN-REG*	Non-Sig	Non-Sig	Non-Sig	DOWN-REG*	Non-Sig	DOWN-REG*
**CYP51A1**	DOWN-REG*	DOWN-REG*	Non-Sig	UP-REG*	Non-Sig	Non-Sig	DOWN-REG*	DOWN-REG*	Non-Sig	UP-REG*	DOWN-REG*	Non-Sig
**DHCR24**	DOWN-REG*	DOWN-REG*	UP-REG*	UP-REG*	Non-Sig	DOWN-REG*	NA	NA	NA	NA	NA	NA
**DHCR7**	DOWN-REG*	DOWN-REG*	UP-REG*	UP-REG*	Non-Sig	Non-Sig	DOWN-REG*	DOWN-REG*	Non-Sig	UP-REG*	DOWN-REG*	Non-Sig
**FDFT1**	Non-Sig	DOWN-REG*	Non-Sig	Non-Sig	Non-Sig	Non-Sig	NA	NA	NA	NA	NA	NA
**FDPS**	DOWN-REG*	DOWN-REG*	UP-REG*	UP-REG*	Non-Sig	Non-Sig	DOWN-REG*	DOWN-REG*	Non-Sig	Non-Sig	DOWN-REG*	DOWN-REG*
**HMGCR**	DOWN-REG*	DOWN-REG*	UP-REG*	UP-REG*	Non-Sig	Non-Sig	DOWN-REG*	Non-Sig	UP-REG*	Non-Sig	Non-Sig	Non-Sig
**HMGCS1**	DOWN-REG*	DOWN-REG*	UP-REG*	UP-REG*	Non-Sig	Non-Sig	DOWN-REG*	DOWN-REG*	UP-REG*	UP-REG*	Non-Sig	Non-Sig
**LSS**	DOWN-REG*	DOWN-REG*	UP-REG*	UP-REG*	Non-Sig	Non-Sig	DOWN-REG*	DOWN-REG*	Non-Sig	Non-Sig	DOWN-REG*	DOWN-REG*
**MVD**	Non-Sig	DOWN-REG*	Non-Sig	UP-REG*	Non-Sig	Non-Sig	NA	NA	NA	NA	NA	NA
**MVK**	DOWN-REG*	DOWN-REG*	Non-Sig	UP-REG*	Non-Sig	Non-Sig	Non-Sig	Non-Sig	Non-Sig	Non-Sig	Non-Sig	DOWN-REG*
**NSDHL**	DOWN-REG*	DOWN-REG*	Non-Sig	UP-REG*	Non-Sig	Non-Sig	DOWN-REG*	DOWN-REG*	Non-Sig	Non-Sig	DOWN-REG*	DOWN-REG*
**PMVK**	Non-Sig	DOWN-REG*	Non-Sig	Non-Sig	Non-Sig	Non-Sig	DOWN-REG*	Non-Sig	Non-Sig	DOWN-REG*	DOWN-REG*	DOWN-REG*
**SQLE**	DOWN-REG*	DOWN-REG*	UP-REG*	UP-REG*	Non-Sig	Non-Sig	DOWN-REG*	Non-Sig	Non-Sig	UP-REG*	DOWN-REG*	DOWN-REG*
**AQP1**	UP-REG*	Non-Sig	Non-Sig	Non-Sig	UP-REG*	Non-Sig	NA	NA	NA	NA	NA	NA
**AQP2**	Non-Sig	UP-REG*	DOWN-REG*	Non-Sig	Non-Sig	Non-Sig	NA	NA	NA	NA	NA	NA
**AQP3**	UP-REG*	Non-Sig	DOWN-REG*	Non-Sig	Non-Sig	Non-Sig	NA	NA	NA	NA	NA	NA
**ATP1B3**	Non-Sig	Non-Sig	DOWN-REG*	DOWN-REG*	Non-Sig	Non-Sig	UP-REG*	Non-Sig	DOWN-REG*	Non-Sig	Non-Sig	Non-Sig
**ENaC**	Non-Sig	Non-Sig	Non-Sig	Non-Sig	Non-Sig	Non-Sig	NA	NA	NA	NA	NA	NA
**KCNJ8**	UP-REG*	UP-REG*	DOWN-REG*	Non-Sig	Non-Sig	Non-Sig	NA	NA	NA	NA	NA	NA
**SLC9A7**	Non-Sig	Non-Sig	Non-Sig	Non-Sig	Non-Sig	Non-Sig	NA	NA	NA	NA	NA	NA

Supplementary Table ST3. RT-qPCR validations. Mean ±SD (n = 5) fold changes for key genes involved in the cholesterol biosynthesis pathway, genes coding for ion transporters and aquaporins in the Arabian camel kidney medulla and cortex. Comparisons between control and dehydration/rehydration were done using ANOVA with Turkey's post hoc test. *** $p_{adj} < 0.001$, ** $p_{adj} < 0.01$, *$p_{adj} < 0.05$.

	**Medulla**						**Cortex**					
	Control		Dehydration Vs. Control		Rehydration Vs. Control		Control		Dehydration Vs. Control		Rehydration Vs. Control	
	**Mean**	**±SD**	**Mean**	**±SD**	**Mean**	**±SD**	**Mean**	**±SD**	**Mean**	**±SD**	**Mean**	**±SD**
**ACAT2**	1.35	1.04	*0.41	0.26	*0.57	0.54	1.98	2.58	1.54	0.93	2.92	2.89
**CYP51A1**	1.24	0.87	*0.42	0.21	0.87	0.70	1.19	0.55	0.82	0.44	1.21	0.37
**DHCR24**	1.12	0.64	***0.2	0.07	*0.68	0.20	1.31	0.90	**0.24	0.24	0.66	0.67
**DHCR7**	1.01	0.16	***0.43	0.09	0.96	0.24	1.01	0.16	**0.49	0.15	1.21	0.56
**FDFT1**	1.11	0.52	0.73	0.26	1.48	0.14	1.01	0.17	0.80	0.28	1.54	1.02
**FDPS**	1.28	0.80	0.87	0.64	0.72	0.31	1.10	0.48	1.11	0.48	0.84	0.27
**HMGCR**	1.13	0.58	***0.38	0.22	**1.75	0.28	1.02	0.20	***0.43	0.11	1.84	1.71
**HMGCS1**	1.30	0.91	**0.42	0.23	0.83	0.35	1.15	0.58	0.75	0.26	1.27	0.34
**LSS**	1.10	0.47	***0.39	0.20	1.42	0.37	1.01	0.18	***0.42	0.14	1.65	1.49
**MVD**	1.08	0.42	*0.51	0.35	0.93	0.47	1.14	0.66	0.74	0.30	1.40	0.43
**MVK**	2.03	2.15	0.45	0.29	0.44	0.33	2.51	3.15	2.04	1.36	5.07	5.21
**NSDHL**	1.34	1.00	*0.38	0.20	*0.54	0.52	2.50	3.60	1.80	1.39	4.14	3.65
**PMVK**	1.40	1.09	*0.37	0.24	*0.44	0.39	1.36	1.03	0.91	0.48	1.24	0.47
**SQLE**	1.03	0.29	***0.34	0.12	0.69	0.46	1.03	0.25	***0.28	0.19	0.83	0.37
**AQP1**	1.06	0.37	0.96	0.48	0.69	0.18	1.05	0.37	*2.13	0.61	1.76	0.86
**AQP2**	1.04	0.27	***2.08	0.53	1.12	0.15	1.12	0.65	1.28	0.26	*0.52	0.28
**AQP3**	1.23	0.98	*2.92	0.86	1.06	0.45	1.09	0.49	***2.74	0.68	1.00	0.38
**ATP1B3**	1.40	0.83	1.84	1.11	1.53	0.74	1.07	0.50	2.25	0.72	1.18	0.39
**ENaC**	1.03	0.25	0.74	0.25	1.13	0.20	1.53	1.44	0.89	0.75	2.57	2.12
**KCNJ8**	1.10	0.48	**2.17	0.97	1.23	0.43	1.03	0.25	**2.19	0.86	**1.00	0.29
**SLC9A7**	1.33	1.05	1.85	0.87	1.07	1.00	1.12	0.40	***1.71	0.80	**1.03	0.60

Supplementary Table ST4. RIN values for the samples used for RNAseq analyses. CC – Cortex Control, CD – Cortex Dehydration, CR – Cortex Rehydration, MC – Medulla Control, MD – Medulla Dehydration, MR – Medulla Rehydration. Mean and SD are shown in blue bottom rows.

Cortex			Medulla		
Sample Name	RIN	28/18S	Sample Name	RIN	28/18S
CC1	7.8	1.6	MC1	8.8	1.6
CC2	7.5	1.7	MC2	9.1	1.6
CC3	8.2	1.8	MC3	8.0	1.4
CC4	8.7	2	MC4	8.4	1.6
CC5	8.4	2	MC5	8.1	2.1
Mean	8.1		Mean	8.5	
SD	0.5		SD	0.5	
CD1	7.6	1.9	MD1	9.0	1.7
CD2	8	1.8	MD2	8.6	1.6
CD3	7.6	1.8	MD3	8.7	1.7
CD4	7.8	1.7	MD4	8.9	1.6
CD5	7.4	1.5	MD5	9.0	1.5
Mean	7.7		Mean	8.8	
SD	0.2		SD	0.2	
CR1	8.5	1.5	MR1	8.6	1.8
CR2	7.9	1.5	MR2	8.9	1.8
CR3	7.1	1.5	MR3	8.0	1.7
CR4	7.1	1.7	MR4	8.9	1.7
CR5	7.5	2	MR5	9.0	1.5
Mean	7.6		Mean	8.7	
SD	0.6		SD	0.4	

Supplementary Table ST5. RT-qPCR oligos. List of transcript-specific primer pairs designed for RT-qPCR gene validation. Primers were designed using Primer-BLAST (NCBI) [131] and an available reference genome of the one-humped Arabian camel (accession No.: PRJNA310822). All oligos were provided by Merck in dry format (Synthesis Scale = 0.025µmol, desalt purification).

		Primer sequences	
	**Gene symbols**	**Forwards**	**Reverse**
**House Keeping Gene**	*PPIA*	5’-ACCACCAGACCATTCCTTCT-3’	5’-TATGGAACCCCGAAAACTGC-3’
**Genes encoding enzymes involved in cholesterol biosynthesis**	*ACAT2*	5’-AGGTTGGTCACTGGAGGATG-3’	5’-AGTTATTGCAGCAGACACGG-3’
	CYP51A1	5’-TGCAAATACCTTATGAGAGTAGCC-3’	5’-TGCCTAGGGCCACCAATAAT-3’
	DHCR7	5’-CGAGTTGGGAAGTGGTTTGA-3’	5’-GCGAAGGACAGGTTGATGAG-3’
	DHCR24	5’-GAAGTGTGCATGGGTTCCAG-3’	5’-GTATAGGGAGCCGTCGAACA-3’
	FDFT1	5’-GATTTGGGATGGCGGAGTTT-3’	5’-CGAGAATAGACGGGAAAGGC-3’
	FDPS	5’-AACGCCATTGGAGGCAAGTA -3’	5’-CCCCATCCTCTCACCAGTTC-3’
	HMGCR	5’-TTGATTGACCTTTCCAGAGCA-3’	5’-AGACATTCCACAAGAGCATCG-3’
	HMGCS1	5’-AAGATGCCACGCCAGGTAAA-3’	5’-AAGCCTGCTCAGTCAACCTTA-3’
	LSS	5’-AGGACTATGGCTGCATCAAT-3’	5’-GGAATGCATACAGTACGTGG-3’
	MVD	5’-GTCAACATCGCGGTCATCAA-3’	5’-GTGACGCTCAGAGAGGAGTT-3’
	MVK	5’-TATGGGAAAGTCGGCCTCAA-3’	5’-CTCGCTCCAGAAAGCTCGT-3’
	NSDHL	5’-AGGCTACACCGTCAATGTCT-3’	5’-ACCCAGAAAGAACTGCACCT-3’
	PMVK	5’-CGGAGAGTGTCTGACATCCA-3’	5’-GTCCAGGCCACATTCTGATTC-3’
	SQLE	5’-AACGCCATTGGAGGCAAGTA-3’	5’-CCCCATCCTCTCACCAGTTC-3’
**Genes encoding ion transporters**	*KCNJ8*	5’-TTACCACGCAAGCAAGAACC-3’	5’-ACAATGGACACGAAGCGATG-3’
	SLC9A7	5’-GCAGACTCTGGACCATCCTA-3’	5’-AAGGGAAGCTTTCCCCTCTTA-3’
	ATP1B3	5’-TGGGTTAGGTAGACGTGTGT-3’	5’-CTCCGTTTGCCATCACCATT-3’
	ENaC	5’-AGCTCCAACCTCTGGATGTC-3’	5’-AAGTCATTCTGCTCTGTGCG-3’
**Gene encoding Aquaporins**	*AQP2*	5’-CTCTAGGGCTGTACTCCTGC-3’	5’- CTAGGCTTGGCTCCTACCTC-3’
	AQP1	5’-CACGGGGTGTGGTATTAACC-3’	5’- CCCACCCAGAAAATCCAGTG-3’
	AQP3	5’-CCTACCCCTCTGGACACTTG-3’	5’-ACAGGGTTGTTGTAGGGGTC-3’
