# Supplementary material for: Multiomic analysis of the Arabian camel (Camelus dromedarius) kidney reveals a role for cholesterol in water conservation
Source: Commun Biol. 2021 Jun 23;4:779. doi: 10.1038/s42003-021-02327-3 (PMC8222267; doi:10.1038/s42003-021-02327-3)
Supplement: Supplementary file 3 — Description of Supplementary Files [file 42003_2021_2327_MOESM3_ESM.pdf]

Description of Additional Supplementary Files

File name: Supplementary Data 1
Description: DESeq2 and DEqMS data. Gene expression and protein abundance data including all identified genes and proteins regardless of significance.

File name: Supplementary Data 2
Description: Gene catalogues of DEGs in the cortex and the medulla under the different conditions.

File name: Supplementary Data 3
Description: Gene Ontology of the DEGs per tissue and condition.

File name: Supplementary Data 4
Description: Gene catalogue of DEGs in cortex and medulla showing the expression profile displayed by each DEG.

File name: Supplementary Data 5
Description: Protein catalogues of DEPs in the cortex and the medulla under the different conditions.

File name: Supplementary Data 6
Description: Gene Ontology of the DEPs per tissue and condition.

File name: Supplementary Data 7
Description: Genes that were differentially expressed at transcript and protein levels.

File name: Supplementary Data 8
Description: Gene Ontology of genes that were differentially expressed at transcript and protein levels.

File name: Supplementary Data 9
Description: Source data for boxplots and statistical analyses.
