# Supplementary material for: Multiomic analysis of the Arabian camel (Camelus dromedarius) kidney reveals a role for cholesterol in water conservation
Source: Commun Biol. 2021 Jun 23;4:779. doi: 10.1038/s42003-021-02327-3 (PMC8222267; doi:10.1038/s42003-021-02327-3)
Supplement: Supplementary file 13 — Reporting Summary [file 42003_2021_2327_MOESM13_ESM.pdf]

nature research

Corresponding author(s): Fernando Alvira-Iraizoz
Abdu Adem

Last updated by author(s): May 19, 2021

Reporting Summary

Nature Research wishes to improve the reproducibility of the work that we publish. This form provides structure for consistency and transparency in reporting. For further information on Nature Research policies, see our Editorial Policies and the Editorial Policy Checklist.

Statistics

For all statistical analyses, confirm that the following items are present in the figure legend, table legend, main text, or Methods section.

n/a	Confirmed	
☐	☒	The exact sample size (n) for each experimental group/condition, given as a discrete number and unit of measurement
☐	☒	A statement on whether measurements were taken from distinct samples or whether the same sample was measured repeatedly
☐	☒	The statistical test(s) used AND whether they are one- or two-sided<br>*Only common tests should be described solely by name; describe more complex techniques in the Methods section.*
☒	☐	A description of all covariates tested
☐	☒	A description of any assumptions or corrections, such as tests of normality and adjustment for multiple comparisons
☐	☒	A full description of the statistical parameters including central tendency (e.g. means) or other basic estimates (e.g. regression coefficient) AND variation (e.g. standard deviation) or associated estimates of uncertainty (e.g. confidence intervals)
☐	☒	For null hypothesis testing, the test statistic (e.g. F, t, r) with confidence intervals, effect sizes, degrees of freedom and P value noted<br>*Give P values as exact values whenever suitable.*
☒	☐	For Bayesian analysis, information on the choice of priors and Markov chain Monte Carlo settings
☒	☐	For hierarchical and complex designs, identification of the appropriate level for tests and full reporting of outcomes
☒	☐	Estimates of effect sizes (e.g. Cohen's d, Pearson's r), indicating how they were calculated

Our web collection on statistics for biologists contains articles on many of the points above.

Software and code

Policy information about availability of computer code

Data collection	No data was collected from other sources
Data analysis	All software and code used to analyze the data has been previously described in the literature and are common, well-established tools used in omics studies. Briefly, An in-house computer (Dell PowerEd geR820 12 core personal supercomputer equipped with 512GB RAM and 12x1TB HDD) was used to process sequencing data using a bespoke pipeline. First, reads were trimmed of adaptor sequences using BBDuk tool followed by FastQC (Andrews et al. 2012) quality control. STAR (Dobin et al. 2013) was used to map reads using default settings to the publicly available Camelus dromedarius genome assembly GCA_000803125.2 (CamDro2) downloaded from the Ensembl 100 database (Yates et al. 2020). Mapped reads were summarized using FeatureCounts (Liao et al. 2014) grouping to gene identifiers. DESeq2 (Love et al. 2014) in Rv3.6.1 (R Core Team (2019)) was used to estimate differential expression of genes between conditions. Proteome Discoverer v2.1 software was used to filter the data with a 5% FDR cut off and remove any contaminants identified. Normalization to total protein content of each sample was performed for the final protein abundance ration. Differential protein expression was calculated using the DEqMS R package (Zhu et al. 2020) using log transformed and median normalized protein abundance ratio values.

For manuscripts utilizing custom algorithms or software that are central to the research but not yet described in published literature, software must be made available to editors and reviewers. We strongly encourage code deposition in a community repository (e.g. GitHub). See the Nature Research guidelines for submitting code & software for further information.

Data

Policy information about availability of data

All manuscripts must include a data availability statement. This statement should provide the following information, where applicable:

- Accession codes, unique identifiers, or web links for publicly available datasets
- A list of figures that have associated raw data
- A description of any restrictions on data availability

The data underlying the transcriptomic analyses, including raw FASTQ files, bulk RNAseq counts, DESeq2 data and project metadata, have been deposited in NCBI's Gene Expression Omnibus 137 and are accessible through GEO Series accession number GSE173683 at https://www.ncbi.nlm.nih.gov/geo/query/acc.cgi?acc=GSE173683. The mass spectrometry proteomics data have been deposited to the ProteomeXchange Consortium via the PRIDE 138 partner repository, and can be accessed with the accession number PXD025644.

Field-specific reporting

Please select the one below that is the best fit for your research. If you are not sure, read the appropriate sections before making your selection.

- [x] Life sciences
- [ ] Behavioural & social sciences
- [ ] Ecological, evolutionary & environmental sciences

For a reference copy of the document with all sections, see nature.com/documents/nr-reporting-summary-flat.pdf

Life sciences study design

All studies must disclose on these points even when the disclosure is negative.

Sample size	The sample size was predetermined based on the number of individuals required for comprehensive RNAseq analysis, which was the primary outcome of this study. Power analysis was carried out using (Busby et al., 2013, Bioinformatics 10.1093/bioinformatics/btt015). The RNAseq analysis was sufficiently powered to reduce the false discovery rate, and to enable systems level analysis (Khang & Lau, 2015, PeerJ 3:e1360).
Data exclusions	Out of the 19 camels included in the experimental protocol, 15 were used for RNA sequencing and peptide quantification. These were randomly selected using the balls in a bag method for groups of more than 5. No data was excluded from further analyses.
Replication	This work was performed using samples from a non-model organism (camel) so replication of the study was not possible.
Randomization	The animals used in these experiments were randomly divided into groups by workers on the camel ranch.
Blinding	The investigators were not blinded during sample collection. An alphanumeric coding system was used post extraction, so investigators performing and analysing RNA sequencing and proteomics datasets were blinded.

Reporting for specific materials, systems and methods

We require information from authors about some types of materials, experimental systems and methods used in many studies. Here, indicate whether each material, system or method listed is relevant to your study. If you are not sure if a list item applies to your research, read the appropriate section before selecting a response.

Materials & experimental systems

n/a	Involved in the study	
x		Antibodies
x		Eukaryotic cell lines
x		Palaeontology and archaeology
	x	Animals and other organisms
x		Human research participants
x		Clinical data
x		Dual use research of concern

Methods

n/a	Involved in the study	
x		ChIP-seq
x		Flow cytometry
x		MRI-based neuroimaging

Animals and other organisms

Policy information about studies involving animals; ARRIVE guidelines recommended for reporting animal research

Laboratory animals	This study did not involved laboratory animals.
Wild animals	This study did not involved wild animals, all dromedary camels were domesticated and raised for human consumption.
Field-collected samples	Nineteen male dromedary camels (Camelus dromedarius) aged 4-5 years, body weight range 276-416 kg, were used in the present

study. The camels were supplied with alfalfa hay as feed and ranch-housed in the United Arab Emirates. Veterinary supervision was provided throughout the experimental period and no signs of distress or illness were identified. After the experimental period, the camels were sacrificed in the local central abattoir for human consumption. Experiments and sampling were carried out in April 2016 in the United Arab Emirates under natural environmental conditions.

Ethics oversight	Samples were shipped frozen on dry ice to the University of Bristol under the auspices of a DEFRA Import Licence (TARP/2016/063). This study was approved by the Animal Ethics Committee of the United Arab Emirates University (approval ID: AE/15/38) and the University of Bristol Animal Welfare and Ethical Review Board

Note that full information on the approval of the study protocol must also be provided in the manuscript.
